# Clinical outcomes in neonates with congenital heart disease with concurrent congenital neurological anomalies

**DOI:** 10.1186/s12887-025-06088-4

**Published:** 2025-10-02

**Authors:** Zareh Torabyan, Hiba El-Rahi, Catherine Y Cong, Ana C. Coll, Laxmi V. Ghimire

**Affiliations:** 1 Department of Pediatrics, University of California, San Francisco, Fresno Campus, Fresno, CA USA; 2 Division of Pediatric Cardiology, Department of Pediatrics, University of California San Francisco, Fresno campus, Fresno, CA USA; 3 Department of Pediatrics, 155 N Fresno St, Fresno, CA 93701 USA

**Keywords:** Congenital heart disease, Central nervous system anomalies, Clinical outcomes, United states, International classification of diseases, Tenth revision

## Abstract

*Introduction*: Children with congenital heart diseases(CHD) may be born with major extracardiac congenital anomalies. We hypothesized that neonates with CHD with concurrent central nervous system congenital lesions (CNS) experience worse clinical outcomes compared to those with isolated CHD. *Methods*: We used the National Inpatient Sample database for the years 2016–2020 to examine the incidence of CHD and CNS lesions among neonates. We further categorized CHD into simple lesions, complex biventricular lesions, and single ventricle lesions. We used descriptive statistics to compare the characteristics of neonates with isolated CHD vs. those with central nervous system anomalies. We then created logistic regression models accounting for important confounding factors to calculate the risk of central nervous system anomalies in outcomes in neonates with CHD. *Results:* We retrieved 564,085 neonates with CHD and found 22,065 with concurrent CNS lesions. In-hospital mortality was significantly higher in combined CHD and CNS cases 9.0%(*n* = 1990) vs. isolated CHD 2.5%, (*n* = 13710), *P* = 0.001. Mortality was higher in those children who underwent surgical repair vs. those without surgical repair for CHD for each category of CHD severity. Major clinical complications were more frequent in cases with CNS involvement. *Conclusions*: Neonates born with CHD and CNS anomalies experience higher morbidity and mortality compared to those with isolated CHD.

## Introduction

Congenital heart disease remains one of the most prevalent and complex congenital anomalies, affecting a significant portion of the global population [1]. Among fetal congenital heart defects (CHD) that have been identified, the greatest portion is attributed to cardiac septal malformations [2]. These anomalies are particularly associated with the most significant extent of connections to chromosomal irregularities and defects beyond the heart [2]. One of the crucial dimensions of CHD is its association with central nervous system (CNS) congenital anomalies. The intricate interplay between cardiac and neural development is increasingly recognized as an essential aspect of comprehensive patient management [3].

The central nervous system plays a pivotal role in coordinating and regulating various bodily functions. The intricate developmental processes that orchestrate both cardiac and central nervous system development occur in parallel during embryogenesis, guided by complex genetic and environmental factors [4]. The initial stages of right ventricle and outflow tract development stem from precursor cells of the cardiac neural crest, which originate in the hindbrain’s posterior region [4, 5]. Variations in the genetic makeup of cell lineages within the second heart field could potentially establish a connection between various congenital heart defects (ventricular septal defects, atrial septal defects, and tetralogy of Fallot) and anomalies in the development of the midbrain and hindbrain [1, 6]. Disruptions at any stage of these intricate developmental pathways can lead to many anomalies, potentially affecting the heart and the central nervous system [1]. Recent research has illuminated the striking correlations between CHD and central nervous system anomalies, ranging from subtle neurodevelopmental deficits to more overt structural abnormalities. In the literature, fetuses with congenital heart defects (CHD), the occurrence of structural brain abnormalities during the prenatal stage was found to be 28% (59/221) [7]. The study reported percentage remained at 25% (10/41) when specifically considering tetralogy of Fallot (TOF) [7]. The same report mentioned that the fetuses affected by CHD were observed to have a higher likelihood of experiencing diminished brain volume, delayed brain maturation, and changes in brain circulation compared to those without CHD [7].

As there is limited information on this topic, we conducted a comprehensive study that systematically assesses the risk factors and complications associated with CNS congenital anomalies among neonates diagnosed with CHD.

## Materials and methods

In this study, we used the nationally representative National Inpatient Sample database, spanning the years 2016–2020. A neonate was defined as any patient aged 28 days or younger. Inclusion required at least one hospital admission during the initial 28‑day window, regardless of whether it was the initial delivery admission or a readmission after discharge. Our analytical approach involved a categorization of CHD into three distinct subtypes: simple lesions, complex bi-ventricular lesions, and single ventricle lesions [8]. Supplemental Table (1) This detailed categorization acknowledged the heterogeneity within the broader spectrum of CHD. We used categorization of congenital central nervous system conditions to include both brain and spine anomalies. Supplemental Table (2) We also stratified and evaluated the outcomes in the neonates who underwent surgical repair for CHD vs. those who did not during the admission.

To identify neonates with congenital heart disease (CHD) and central nervous system anomalies, we utilized the International Classification of Diseases, Tenth Revision, Clinical Modification codes. For CHD we used International Classification of Diseases-10 codes Q20-Q26 and for central nervous system anomalies we used codes Q00- Q07 and further divided into brain, spinal cord and combined anomalies. ICD-10 codes Q00–Q01 encompass major neural tube defects, including anencephaly and encephalocele. Q02 designates microcephaly, while Q03 refers to congenital hydrocephalus. Q04 includes a range of other cerebral malformations, such as lissencephaly and holoprosencephaly. Q05 and Q06 cover spina bifida and additional spinal cord anomalies, and Q07 captures other specified congenital disorders of the nervous system. Supplemental Table 2 We excluded the neonates with chromosomal anomalies and known genetic conditions. Simple or mild CHD encompasses conditions such as atrial septal defect, ventricular septal defect, patent ductus arteriosus, and other septal defects. Single ventricle lesions consisted of hypoplastic left heart syndrome, tricuspid atresia/stenosis, aortic atresia/stenosis, and common ventricle. The remaining lesions were classified as biventricular complex lesions. Supplementary Table 1.

To evaluate baseline characteristics, we employed descriptive statistics to compare the characteristics of neonates presenting with isolated CHD against those with concurrent CNS anomalies.

Our primary objective was to describe disparities in mortality rates between neonates with isolated CHD and those presenting with concurrent CNS anomalies for both surgical and non-surgical neonates in each complexity of CHD category. Our secondary objectives entails a comparative analysis of associated clinical complications.

The software tools Stata version 15 [9] and RStudio [10] were used for data analysis and statistical modeling. We used *ggplot* for figures [11].

## Results

The study retrieved data on 564,085 neonates diagnosed with CHD, out of which 22,065 (4%) had concomitant CNS congenital lesions (Table 1). There were 34,865 (6.2%) CHD surgical cases in total and 2,095 (9.5%) of neonates with CNS anomalies had CHD surgery. Mortality for the CHD neonate with CNS anomalies who underwent surgical repair was 13.6% (*n* = 285). The in-hospital mortality rate was 2.5% (*n* = 13,710) for isolated CHD compared to 9.0% (*n* = 1,990) for those with combined CHD and central nervous system anomalies, (*P* < 0.001).

**Table 1:** Categories of congenital heart disease with central nervous system anomalies

CHD	CNS anomalies		Total
	No	Yes	
Simple CHD	449,375 (96.3%)	17,320 (3.7%)	466,695
Complex biventricular CHD	83,025 (95.2%)	4180 (4.8%)	87,205
Single ventricle CHD	9620 (94.5%)	565 (5.5%)	10,185
Total	542,020 (96.1%)	22,065 (3.9%)	564,085

*P* < 0.001

*CHD* congenital heart disease

*CNS* central nervous system

We further analyzed the mortality in CHD patients with and without CNS anomalies in different CHD categories. We found that for each category of CHD, the mortality was higher in neonates with concomitant CNS lesions. Table 2. Specifically, mortality was 27%(*n* = 105) in CHD/CNS neonates compared to 13%(*n* = 1015) in those with isolated CHD lesions in those who underwent surgical repair for CHD. Table 2.

**Table 2:** Mortality in neonatal cardiac surgery in CHD patients with and without CNS anomalies in different categories of CHD

CHD categories	CHD	CHD and CNS	*P*-value
Simple CHD	7%(440/6230)	12%(80/640)	< 0.001
Complex biventricular CHD	5%(960/18,965)	9%(100/1060)	< 0.001
Single ventricle CHD	13%(1015/7550)	27%(105/390)	< 0.001

Regarding other serious clinical complications, neonates with CHD and concomitant CNS congenital lesions(combined surgical and non-surgical cohort) exhibited higher risks compared to those with isolated CHD. The rates of respiratory failure (0.6% vs. 0.3%, *p* < 0.004), acute kidney injury (4.8% vs. 2.3%, *p* < 0.001), and sudden cardiac arrests (0.2% vs. 0.1%, *p* = 0.025) were all significantly elevated in the former group (Table 3). Within the surgical subgroup, ECMO was more often require in CHD neonates with concomitant CNS lesions compared to isolated CHD lesions, 2.1% vs. 3.6%, *P* = 0.038. Table 4 and 5.

**Table 3:** Clinical complications in neonates with CHD and concomitant central nervous system lesions in combined cohort (surgical and non-surgical)

Complications	CNS anomalies (*n*, %)		*P*-value
	No	Yes	
Resp failure	1885 (0.3%)	135 (0.6%)	0.004
Acute kidney injury	12,505 (2.3%)	1050 (4.8%)	< 0.001
ECMO	1490 (0.3%)	95 (0.4%)	0.064
Tachyarrhythmias	11,355 (2.1%)	530 (2.4%)	0.179
Heart block	3660 (0.7%)	200 (0.9%)	0.075
Sudden cardiac arrest	530 (0.1%)	45 (0.2%)	0.025
In-hospital mortality	13,710 (2.5%)	1990 (9.0%)	< 0.001

ECMO = extracorporeal membrane oxygenation

**Table 4:** Clinical complications in neonates with CHD and concomitant central nervous system lesions in those who underwent surgical procedure to repair CHD. Total patients = 2095 in CNS + CHD and 32,770 in isolated CHD

Complications	CNS anomalies (*n*, %)		*P*-value
	No	Yes	
Resp failure	780(2.3%)	60(2.9%)	0.53
Acute kidney injury	4555(13.9%)	300(14.3%)	0.81
ECMO	675(2.1%)	75(3.6%)	0.038
Tachyarrhythmias	3640(11.1%)	240(11.5%)	0.82
Heart block	1460(4.4%)	110(5.2%)	0.44
Sudden cardiac arrest	295(0.9%)	25(1.2%)	0.54
In-hospital mortality	2415(7.4%)	285(13.6%)	< 0.001

ECMO = extracorporeal membrane oxygenation

In a subpopulation of individuals with CHD, the prevalence of CNS anomalies was assessed. There were 19,230 cases of congenital brain anomalies, 2400 cases of congenital spinal cord anomalies, and 1095 cases of combined CNS anomalies (Table 6). Among individuals with CHD, mortality was 3% of those without brain anomalies vs. 10% with brain anomalies *P* < 0.001 (Table 6). For spinal cord anomalies, neonatal mortality was 5% in those with CHD lesions, *P* = 0.0012.

**Table 5:** Clinical complications in neonates with CHD and concomitant central nervous system lesions in those in non- surgical groups. Total patients=19,970 in CNS+CHD and 509,040 in isolated CHD

Complications	CNS anomalies (n, %)		P-value
	No	Yes	
Resp failure	1105(0.21%)	75(0.4%)	0.037
Acute kidney injury	7950(1.6%)	750(3.8%)	<0.001
ECMO	815(0.16%)	20(0.1%)	0.34
Tachyarrhythmias	7715(1.51%)	290(1.5%)	0.75
Heart block	2200(0.43%)	90(0.5%)	0.86
Sudden cardiac arrest	235(0.05%)	20(0.1%)	0.12
In-hospital mortality	11,295(2.2%)	1705(8.5%)	<0.001

**Table 6:** In-hospital mortality of CHD patients by CNS lesion type

Mortality Status	Anomaly Status	Brain Anomalies*	Spinal Cord Anomalies^#^	Combined CNS Anomalies^&^
Survivors	No Anomalies	530,765 (97%)	545,870 (97%)	547,125 (97%)
	With Anomalies	17,380 (90%)	2275 (95%)	1020 (93%)
In-hospital mortality	No Anomalies	13,850 (3%)	15,575 (3%)	15,625 (3%)
	With Anomalies	1850 (10%)	125 (5%)	75 (7%)
Total Cases		563,845	563,845	563,845

*P < 0.001

^#^ *P* = 0.0012

^&^*P* = 0.0003

## Discussion

This study explores the impact of congenital central nervous system anomalies on morbidity and mortality in patients with congenital heart disease (CHD). Our findings reveal a strong correlation between CHD severity, the presence of CNS, and clinical outcomes, providing valuable insights for neonatal care.

Among affected individuals, congenital brain anomalies were most common, followed by spinal cord anomalies and combined central nervous system anomalies. The presence of these conditions was associated with significantly lower survival rates during hospitalization—patients with brain anomalies had a 10% lower survival rate, while those with spinal cord or combined central nervous system anomalies had a 5% and 7% lower survival rate, respectively.

Neonates with both CHD and central nervous system anomalies faced higher morbidity and mortality, likely due to the compounded effects of two major system abnormalities. Previous reports have explained that the fetuses with CHD exhibited reduced brain volume, delayed maturation, and altered circulation, often with a lower middle cerebral artery pulsatility index and cerebroplacental ratio [6], which could affect the clinical outcomes.

The analysis of in-hospital mortality revealed a clear trend—mortality rates increased with CHD complexity, emphasizing the importance of considering both CHD severity and central nervous system anomalies in risk assessment. Additionally, neonates with both conditions experienced higher rates of complications, including respiratory failure, acute kidney injury, and sudden cardiac arrest, underscoring the need for comprehensive monitoring, management strategies, and resource allocation for this vulnerable population.

When comparing mortality and complications in the neonates who underwent surgical repair for CHD, those with concomitant congenital CNS lesions had higher mortality and complications compared to those with isolated CHD lesions for each category of CHD lesions. This will help in risk categorizing the neonates and help intensive care unit physicians and surgical teams in counseling families while planning surgical repair of these lesions.

While this study benefits from the use of a nationwide database, several limitations must be acknowledged. First, neonates were identified using ICD-10 codes. Our analysis was restricted to neonates with congenital heart disease (CHD) and central nervous system anomalies, excluding infants and older children. Our data source does not consistently capture clinical encounters beyond 28 days of life; therefore, cases presenting later were not classified as neonatal. We acknowledge that this approach likely enriches the cohort for infants requiring earlier intervention and may underrepresent milder lesions, such as isolated secundum atrial septal defects. Additionally, the cross-sectional nature of our study limits our ability to assess the trajectory of outcomes over time, preventing a comprehensive understanding of both short-term and long-term implications.

Furthermore, despite the heterogeneity within CHD, we categorized it into three groups based on previously established criteria [8]. Supplementary Table 1. We recognize that the anatomic and physiologic heterogeneity of congenital heart disease means any grouping will have limitations. To facilitate our sub-analysis, we chose to use widely accepted classifications, understanding that some variability inevitably remains. Depending on lesion severity and associated anatomy, certain defects may ultimately require single‐ventricle palliation. Thus, there is an inherent uncertainty in classification, and individual patient factors often guide the final management pathway.

Another key limitation is the lack of certain clinical variables within the NIS database. Important variables such as medication use, left ventricular function on echocardiography, and details related to congenital heart surgeries—including bypass and cross-clamp times—are not available. Additionally, databases like NIS may incompletely reflect patients’ clinical courses. However, we attempted to mitigate this limitation by adjusting for patient characteristics in our analysis.

## Conclusion

Neonates born with the dual challenge of congenital heart disease (CHD) and central nervous system (CNS) anomalies often confront heightened levels of morbidity and mortality in comparison to their counterparts with isolated CHD. This study’s crucial insights into the increased health risks associated with the concurrent presence of CHD and central nervous system anomalies have widespread implications especially those who undergo surgical repair for CHD. The findings not only serve as a valuable resource for clinicians but also empower family members and payers with essential information for effective counseling and strategic planning to optimize patient care. The enhanced understanding of these complex interactions aids in formulating more informed and tailored healthcare strategies, ensuring a comprehensive and compassionate approach to the management of neonates facing the intricate challenges posed by both CHD and CNS congenital anomalies.

## Data Availability

No datasets were generated or analysed during the current study.

## Abbreviations

CHD: congenital heart disease
CNS: central nervous system
